# Can medical residency keep young specialists in the place where they graduate? Cross-sectional and exploratory study of the first seven years after implementation of medical residency programs in the State of Tocantins, Brazil

**DOI:** 10.1590/1516-3180.2021.0731.R1.14122021

**Published:** 2022-08-29

**Authors:** Giovanna Tandaya Grandi, Raquel Prudente de Carvalho Baldaçara, Itágores Hoffman I Lopes Sousa Coutinho, Leonardo Baldaçara

**Affiliations:** I Undergraduate Student, Universidade Federal do Tocantins, Palmas (TO), Brazil.; II MD, PhD. Assistant Professor, Health Department of the State of Tocantins, Universidade Federal do Tocantins (UFT), Palmas (TO), Brazil.; III MD, PhD. Assistant Professor, Health Department of the State of Tocantins, Universidade Federal do Tocantins (UFT), Palmas (TO), Brazil.; IV MD, PhD. Assistant Professor, Health Department of the State of Tocantins, Universidade Federal do Tocantins (UFT), Palmas (TO), Brazil.

**Keywords:** Internship and residency, Education, medical, Employment, Physicians, Public health, Work, Clinical competence, Postgraduation, Medical residents, Clinical skill, Human

## Abstract

**BACKGROUND:**

New medical schools and new medical residencies in Brazil, mainly in its interior, were opened under the justification of collaborating towards distribution of these healthcare professionals and specialist doctors across the national territory. However, this proposal did not guarantee that medical practitioners would become established in the place where they graduated and specialized.

**OBJECTIVE:**

To calculate, through interviews, how many specialists who graduated in the state of Tocantins stayed there after finishing their medical residency; and to analyze the factors that made them stay or leave the place.

**DESIGN AND SETTING:**

Cross-sectional exploratory study conducted at a Brazilian federal public higher education institution.

**METHODS:**

All graduates from medical residencies in Tocantins, who graduated between 2013 and 2019, were contacted by telephone and, after obtaining consent, an interview was conducted. The interviews took place between June 2020 and January 2021.

**RESULTS:**

The permanence of medical residency graduates in the state increased from 50% in an earlier study to 55.8% in the current study, thus showing a situation of stability. In addition, we detected some reasons for staying or not. In a multivariate analysis, only working in the state capital was related to staying in the state of Tocantins, showing a 5.6 times greater chance.

**CONCLUSIONS:**

The percentage of those who remained was just over 50%, even some years after implementation of the first programs. Most specialists remained working for the state health department, with a smaller proportion in municipal health departments, and were concentrated in the state capital.

## INTRODUCTION

According to the Medical Demographic Census of 2020,^1^ Brazil has reached the goal of 500,000 doctors. This accelerated increase has been due to the opening of new medical graduation opportunities and increased vacancies within medicine.^1^ However, the rising number of physicians in this country has not guaranteed egalitarian geographical distribution. Nor has it ensured the qualifications that are necessary for the needs of the population to be met.^1^

Medical residency is a type of postgraduate education for doctors that ensures ethical and professional qualification. It is the “gold standard” of specialization.^2^ Through this qualification, residents get the title of specialist in a medical field. However, for the specialization to be considered “medical residency”, it needs to be accredited by the National Committee of Medical Residency.^2,3^ Despite the increase in medical graduations, the distribution of the number of residencies and the number of specialist physicians remains uneven among the various regions and states in Brazil.^2,3^

While this country has an average ratio of 2.27 doctors per thousand inhabitants, the northern region has a ratio of 1.30, i.e., 43% lower than the national average ratio. Likewise, in the northeastern region, the ratio is 1.69.^2^ The same is seen in relation to the proportion of specialists over general practitioners in the northern region of Brazil, which is the smallest in the country (1.08), and the highest concentration of specialists in this region is in state capitals.^2^

New medical schools in Brazil, especially in its interior, were opened under the justification of collaborating with the distribution of these healthcare professionals and specialist doctors across the national territory. However, this proposal did not guarantee that medical practitioners would become established in the place where they graduated and specialized.^4^

A pilot study conducted in the state of Tocantins that analyzed the prevalence of remaining in the state between 2013 and 2017 revealed that 65.9% of residents stayed in Tocantins.^5^ Another complete survey considering only the years 2013 and 2014 revealed that 50% of graduating residents remained in the state.^3^

These data demonstrated that medical residents’ decisions on where to settle are based on numerous factors that go beyond the increase in vacancies, such as working conditions, sex, remuneration, city of origin, family and proximity to large urban and economic centers.^3^ Therefore, there is a need to analyze the reasons that lead graduates to leave the state so that, in addition to expansion of opportunities, other aspects can be considered in order to increase the proportion of medical residents who stay.

Thus, the aim of the current study was to analyze how many residents who concluded their first medical residency between 2013 and 2019 remained in the state of Tocantins after finishing. We tried to find factors that related to whether they stayed or not.

## OBJECTIVE

Residents who graduated in the state of Tocantins were interviewed to ask them whether they had stayed in the state after finishing medical residency, with the aim of analyzing the factors that influenced their decision to stay or leave the state.

## METHODS

This was an observational and exploratory cross-sectional study in which all graduates from medical residency in Tocantins, in the years 2013 to 2019, were evaluated. The subjects were contacted by telephone to obtain consent and then to answer the questions. Subjects were excluded if they did not agree to take part or could not be found. The period over which the interviews took place was from June 2020 to December 2020.

The variables (questions asked) were the following: year of graduation; residency program attended; age; date of birth; gender; marital status; whether before the residency they had worked in Tocantins or in any municipal network in this state; whether before the residency they worked in the public network of the state of Tocantins; whether after graduation they stayed in the state of Tocantins and the reason for staying or leaving; the current state where they were living; whether they were still working in the specialty in which they graduated; whether they were currently working in a public state network in another state or in Tocantins; whether they were currently working in a municipal network and in which city; whether they were currently working in the private network of Tocantins; whether their current income was higher than their residency income; hours worked weekly; whether the residency had improved their medical practice; and whether they had subsequently done another medical residency, and if so, in what specialty and where.

The interviews took place via telephone. Out of the 265 graduates contacted, 240 agreed to take part and completed the survey. Nine graduates were not found, fourteen were found but did not respond to the messages, and two refused to take part in the interview. This study was submitted to and approved by the research ethics committee of the Federal University of Tocantins, under the number CAAE 73833615.5.0000.5519; date: September 22, 2017.

### Statistical analysis

The data are presented as numbers and percentages. We compared ages by means of the Kruskal-Wallis test and other nominal variables using the chi-square test. Significance was set at α ≤ 0.05. The main outcome was whether the doctor stayed in the state of Tocantins. We compared this outcome (dependent) with other variables as factors, using a general linear model with a logistic regression tool. In a multivariate analysis, we adjusted the significance using the Bonferroni correction for α ≤ 0.0008.

## RESULTS

Details of the sample are presented in Table 1.

**Table 1:** Main variables according to years of conclusion of residency. Comparisons made using the Kruskal-Wallis and chi-square tests

Variable		2013 n = 15	2014 n = 21	2015 n = 24	2016 n = 34	2017 n = 40	2018 n = 51	2019 n = 55	Total	Z/χ^2^, P
**Age (mean** ± **standard deviation)**		36.7 ± 3.4	35.5 ± 3.5	35.4 ± 4.0	35.6 ± 5.7	34.6 ± 6.9	32.3 ± 3.5	32.5 ± 4.6	**34.1** ± **5.0**	**45.40, P < 0.01**
**Up to 30 years of age**		–	–	–	1 (2.9%)	11 (27.5%)	15 (29.4%)	23 (41.8%)	**50 (20.8%)**	
**From 31 to 40 years of age**		13 (86.7%)	17 (81.0%)	22 (91.7%)	29 (85.3%)	23 (57.5%)	34 (66.7%)	26 (47.3%)	**164 (68.3%)**	**45.30, P < 0.01**
**More than 40 years of age**		2 (13.3%)	4 (19.0%)	2 (8.3%)	4 (11.8%)	6 (15.0%)	2 (3.9%)	6 (10.9%)	**26 (10.8%)**	
**Gender**										
	Male	7 (46.7%)	9 (42.9%)	7 (29.2%)	11 (32.4%)	14 (35.0%)	16 (31.4%)	19 (34.5%)	**83 (34.6%)**	**2.22, 0.90**
	Female	8 (53.3%)	12 (57.1%)	17 (70.8%)	23 (67.6%)	26 (65.0%)	35 (68.6%)	36 (65.5%)	**157 (65.4%)**	
**Institution**										
	Universidade Federal do Tocantins	15 (100%)	21 (100%)	22 (91.7%)	28 (82.4%)	31 (77.5%)	34 (66.7%)	38 (69.1%)	**189 (78.8%)**	**24.81, 0.02**
	State Health Department of Tocantins	0	0	2 (8.3%)	2 (5.9%)	4 (10.0%)	5 (11.8%)	3 (5.5%)	**17 (7.1%)**	
	Municipal Health Department of Palmas	0	0	0	4 (11.8%)	5 (12.5%)	11 (21.6%)	14 (25.5%)	**34 (14.2%)**	
**Marital status**										
	Single	3 (20%)	8 (38.1%)	5 (20.8%)	4 (11.8%)	11 (27.5%)	23 (45.1%)	22 (40.0%)	**76 (31.7%)**	**25.60, 0.11**
	Married	11 (73.3%)	12 (57.1%)	17 (70.8%)	25 (73.5%)	28 (70.0%)	26 (51.0%)	30 (54.5%)	**149 (62.1%)**	
	Divorced	1 (6.7%)	1 (4.8%)	1 (4.2%)	5 (14.7%)	1 (2.5%)	2 (3.9%)	2 (3.6%)	**13 (5.4%)**	
	Widower	0	0	1 (4.2%)	0	0	0	1 (1.8%)	**2 (0.8%)**	
**Worked in public network before residency**										
	No	5 (33.3%)	5 (23.8%)	4 (16.7%)	13 (38.2%)	12 (30.0%)	19 (37.3%)	14 (25.5%)	**72 (30.0%)**	**5.41, 0.49**
	Yes	10 (66.7%)	16 (76.2%)	20 (83.3%)	21 (61.8%)	28 (70.0%)	32 (62.7%)	41 (74.5%)	**168 (70.0%)**	
**Worked in private network before residency**										
	No	11 (73.3%)	19 (90.5%)	15 (62.5%)	29 (85.3%)	34 (85.0%)	39 (76.5%)	45 (81.8%)	**192 (80.0%)**	**8.18, 0.22**
	Yes	4 (26.7%)	2 (9.5%)	9 (37.5%)	5 (14.7%)	6 (15.0%)	12 (23.5%)	10 (18.2%)	**48 (20.0%)**	
**Stayed in state of Tocantins?**										
	No	4 (26.7%)	13 (61.9%)	11 (45.8%)	10 (29.4%)	19 (47.5%)	26 (51.0%)	23 (41.8%)	**106 (44.2%)**	**8.83, 0.18**
	Yes	11 (73.3%)	8 (38.1%)	13 (54.2%)	24 (70.6%)	21 (52.5%)	25 (49.0%)	32 (58.2%)	**134 (55.8%)**	
**If settled down in Tocantins, why?**										
	Family matters	4 (26.7%)	3 (14.3%)	10 (76.9%)	14 (58.3%)	12 (57.1%)	15 (60.0%)	20 (62.5%)	**78 (58.2%)**	**36.29, 0.20**
	Opportunities in jobs	3 (20.0%)	3 (14.3%)	1 (7.7%)	5 (20.8%)	4 (19.0%)	5 (20.0%)	8 (25.0%)	**29 (21.6%)**	
	Demand for specialty	2 (13.3%)	0	0	3 (12.5%)	1 (4.8%)	0	0	**6 (4.5%)**	
	Local identification/quality of life	2 (13.3%)	1 (4.8%)	2 (15.4%)	1 (4.2%)	4 (19.0%)	1 (4.0%)	2 (6.3%)	**13 (9.7%)**	
	Another residency in the state	0	1 (4.8%)	0	0	0	4 (16.0%)	2 (6.3%)	**7 (5.2%)**	
	Work in a field other than medicine	0	0	0	1 (4.2%)	0	0	0	**1 (0.7%)**	
**If did not settle down in Tocantins, why?**										
	Family matters	0	2 (15.4%)	3 (27.3%)	2 (20.0%)	3 (15.8%)	4 (15.4%)	1 (4.3%)	**15 (14.2%)**	**16.97, 0.52**
	Issues related to jobs	1 (25.0%)	7 (53.8%)	4 (36.4%)	6 (60.0%)	11 (57.9%)	10 (38.5%)	15 (65.2%)	**54 (50.9%)**	
	Another residency	3 (75.0%)	4 (30.4%)	4 (36.4%)	1 (10.0%)	5 (26.3%)	11 (42.3%)	5 (21.7%)	**33 (31.1%)**	
	Other reasons	0	0	0	1 (10.0%)	0	1 (3.8%)	2 (8.7%)	**4 (3.8%)**	
**Current municipality**										
	Palmas	11 (73.3%)	8 (38.1%)	7 (29.2%)	17 (50.0%)	19 (47.5%)	22 (43.1%)	29 (52.7%)	**113 (47.1%)**	**18.24, 0.11**
	Other	1 (6.7%)	3 (14.3%)	2 (8.3%)	4 (11.8%)	3 (7.5%)	6 (11.8%)	2 (3.7%)	**21 (8.7%)**	
	Out of state	3 (20.0%)	10 (47.6%)	15 (62.5%)	13 (38.2%)	18 (45.0%)	23 (45.1%)	24 (43.6%)	**106 (44.2%)**	
**Still works in the field of the specialization**										
	No	4 (26.7%)	7 (33.3%)	10 (41.7%)	6 (17.6%)	7 (17.5%)	15 (29.4%)	7 (12.7%)	**56 (23.3%)**	**11.67, 0.07**
	Yes	11 (73.3%)	14 (66.7%)	14 (58.3%)	28 (82.4)	33 (82.5%)	36 (70.6%)	48 (87.3%)	**184 (76.7%)**	
**Hours worked weekly**										
	Up to 60 hours	4 (26.7%)	10 (47.6%)	4 (16.7%)	11 (32.4%)	13 (32.4%)	15 (29.4%)	17 (30.9%)	**74 (30.8%)**	**5.29, 0.51**
	More than 60 hours	11 (73.3%)	11 (52.4%)	20 (83.3%)	23 (67.6%)	23 (67.6%)	36 (70.6%)	38 (69.1%)	**166 (69.2%)**	
**Bigger salary than in residency**										
	No	0	0	0	0	3 (7.5%)	5 (10.0%)	7 (12.7%)	**15 (6.3%)**	**11.50, 0.07**
	Yes	15 (100.0%)	21 (100.0%)	24 (100.0%)	34 (100.0%)	37 (90.0%)	46 (90.0%)	48 (87.3%)	**225 (93.7%)**	
**Works in a municipal network in the current state**										
	No	13 (86.7%)	16 (76.2%)	19 (79.2%)	20 (58.8%)	26 (65.0%)	38 (74.5%)	36 (65.5%)	**168 (70.0%)**	**15.84, 0.60**
	In the capital	2 (13.3%)	2 (9.5%)	2 (8.3%)	8 (23.5%)	9 (22.5%)	5 (9.8%)	12 (21.8%)	**40 (16.7%)**	
	Another city	0	1 (4.8%)	2 (8.3%)	3 (8.8%)	0	2 (3.9%)	3 (5.5%)	**11 (4.6%)**	
	Out of state	0	2 (9.5%)	1 (4.1%)	3 (8.8%)	5 (12.5%)	6 (11.8%)	4 (7.3%)	**21 (8.8%)**	
**Currently works in a private network**										
	No	0	3 (14.3%)	2 (8.3%)	6 (17.6%)	13 (32.5%)	21 (41.2%)	21 (38.2%)	**66 (27.5%)**	**7.64, 0.27**
	Yes	15 (100%)	18 (85.7%)	22 (91.7%)	28 (82.4%)	27 (67.5%)	30 (58.8%)	34 (61.8%)	**174 (72.4%)**	
**Currently works in a state health department**										
	No	11 (73.3%)	15 (71.4%)	18 (75.0%)	20 (58.8%)	23 (57.5%)	35 (68.6%)	30 (54.2%)	**152 (63.3%)**	**27.55, <0.01**
	Yes	4 (26.7%)	6 (28.6%)	6 (25.0%)	14 (41.2%)	17 (42.5%)	16 (31.4%)	25 (45.5%)	**88 (36.7%)**	
**Specialty**										
	Internal medicine	6 (40%)	5 (23.8%)	6 (25.0%)	7 (20.6%)	10 (25.0%)	8 (15.7%)	5 (9.1%)	**47 (19.6%)**	**89.27, 0.67**
	Family and community medicine	0	1 (4.8%)	0	5 (14.7%)	8 (20.0%)	17 (33.3%)	19 (34.5%)	**50 (20.8%)**	
	Orthopedics and traumatology	0	0	0	0	0	0	2 (3.6%)	**2 (0.8%)**	
	Anesthesiology	0	0	2 (8.3%)	2 (5.9%)	3 (7.5%)	4 (7.8%)	3 (5.5%)	**14 (5.8%)**	
	General surgery	4 (26.7)	6 (28.6%)	6 (25.0%)	6 (17.6%)	6 (15.0%)	6 (11.8%)	5 (9.1%)	**39 (16.3%)**	
	Vascular surgery	0	0	1 (4.2%)	1 (2.9%)	1 (2.5%)	1 (2.0%)	1 (1.8%)	**5 (2.1%)**	
	Dermatology	0	0	0	0	0	0	1 (1.8%)	**1 (0.4%)**	
	Gynecology and obstetrics	0	2 (9.5%)	3 (12.5%)	4 (11.8%)	2 (5.0%)	2 (3.9%)	4 (7.3%)	**17 (7.1%)**	
	Infectiology	0	0	0	0	0	0	1 (1.8%)	**1 (0.4%)**	
	Pediatric intensive care medicine	0	0	0	0	1 (2.5%)	0	1 (1.8%)	**2 (0.8%)**	
	Neonatology	0	0	0	0	0	2 (3.9%)	2 (3.6%)	**4 (1.7%)**	
	Pediatrics	5 (33.3%)	7 (33.3%)	6 (25.0%)	6 (17.6%)	6 (15.0%)	6 (11.8%)	5 (9.1%)	**41 (17.1%)**	
	Psychiatry	0	0	0	0	1 (2.5%)	0	2 (3.6%)	**3 (1.3%)**	
	Angioradiology	0	0	0	1 (2.9%)	1 (2.5%)	1 (2.0%)	1 (1.8%)	**4 (1.7%)**	
	Digestive system surgery	0	0	0	1 (2.9%)	0	1 (2.0%)	1 (1.8%)	**3 (1.3%)**	
	Intensive medicine	0	0	0	0	0	2 (3.9%)	1 (1.8%)	**3 (1.3%)**	
	Rheumatology	0	0	0	1 (2.9%)	1 (2.5%)	1 (2.0%)	1 (1.8%)	**4 (1.7%)**	
**Did residency improve your medical practice?**										
	No	0	0	0	0	0	0	1 (1.8%)	**1 (1.8%)**	**3.38, 0.76**
	Yes	15 (100%)	21 (100%)	24 (100%)	34 (100%)	40 (100%)	51 (100%)	54 (98.2%)	**239 (99.6%)**	

Regarding the institutions from which the subjects graduated, the number increased over the years. All of the 15 and 21 residencies available in 2013 and 2014, respectively, were at the Universidade Federal de Tocantins (UFT). In 2015, in addition to 24 residencies (91.7%) at UFT, there were two (8.3%) at the State Health Department of Tocantins (SHD-TO). In 2016, there were 34 training residencies: 28 (82.4%) at UFT, two (5.9%) at SHD-TO and 4 (11.8%) at the Municipal Health Department of Palmas (MHD). In 2017, there were 40 resident physicians: 31 (77.5%) at UFT, four (10.0%) at SHD-TO and five (12.5%) at MHD. In 2018, 51 resident physicians graduated: 34 (66.7%) from UFT, five (11.8%) from SHD-TO and 11 (21.6%) from MHD. In the last year analyzed (2019), out of a total of 55 residents, 38 (69.1%) were at UFT, three (5.5%) were at SHD-TO and 14 (25.5%) were at MHD. These data show that in comparison with 2013-2014, the number of trained resident doctors increased from 36 to 240, among whom UFT contributed 78.8% of the total, SHD-TO 7.1% and MHD 14.2%.

Considering sex, females represented 66.5% of the total. In 2013, eight residents (53.3%) were women and seven (46.7%) were men. In 2014, 12 (57.1%) were women and nine (42.9%) were men. In 2015 there was the greatest difference in all the years of this study, with 17 women (70.8%) and six men (29.2%). In 2016, there were 23women (67.6%) and 11 men (32.4%). In 2017, 26 (65.0%) were women and 14 (35.0%) were men. In 2018, 35 (68.6%) were women and 16 (31.4%) were men. Lastly, in 2019, 36 (65.5%) were women and 19 (34.5%) were men.

Regarding marital status, the majority were married (149; 62.1%), while 76 (31.7%) were single. For more details according to year, and for other marital statuses, see Table 1.

The study results revealed that 168 subjects (70.0%) had worked in the public network before residency, as follows, according to year: 10 (66.7%) of the 2013 residency graduates; 16 (76.2%) from 2014; 20 (83.3%) from 2015; 21 (61.8%) from 2016; 28 (70.0%) from 2017; 32 (62.7%) from 2018; and 36 (74.5%) from 2019. In total, 72 (30.0%) had not worked in a public network before their residency.

When the subjects were asked whether they had worked in a private network before the residency, the majority answered “no” (192; 80%). The highest numbers were presented over the last three years: 45 (81.8%) from 2019, 39 (76.5%) from 2018 and 34 (85.0%) from 2017. However, 2014 presented the highest proportion that answered “no”, with 19, standing for 90.5%; while there were 11 (73.3%) from 2013, 15 (62.5%) from 2015 and 29 (85.3%) from 2016. Regarding the subjects who stayed in the state, the majority answered “yes” (134; 55.8%). Analysis on the numbers over the years showed that these started at 11 (73.3%) in 2013 and 8 (38.1%) in 2014, and increased to 13 (54.2%) in 2015, 24 (70.6%) in 2016, 21 (52.5%) in 2017 and 25 (49.0%) in 2018, respectively. In 2019 the number rose again to 32 (58.2%).

Among those who stayed in the state, the following reasons aided the subjects in making their decision to stay: 78 (58.2%) cited family reasons, 29 (21.6%) job opportunities, six (4.55%) the demand for the specialty, 13 (9.7%) identified with the region and its quality of life, seven (5.2%) wanted to do another residence and one (0.7%) wanted to work in a field other than medicine. On the other hand, a total of 106 (44.2%) left the state, for the following reasons: 54 (50.9%) reported issues relating to job conditions, 33 (31.1%) wanted to do another residency, 15 (14.2%) cited family matters and four (3.8%) had other reasons. For more details, see Table 1.

Regarding the place where the subjects were currently living, 114 (47.5%) stayed in Palmas, the state capital of Tocantins. Proportionally, 2013 had the highest rate, with 11 (73.3%), but in absolute numbers 2019 had the highest number, with 29 (52.7%). In second position, 106 (44.2) were currently living in another state and 21 (8.7%) were currently living in the state of Tocantins, outside of the state capital.

Regarding the subjects’ current field of work, a total of 184 (76.7%) were still working in the specialty corresponding to their medical residency, but 56 (23.3%) said that they were not. In total, 168 (70.0%) did not work in a municipal network, 40 (16.7%) were working in the municipal network of the state capital of Tocantins, 11 (4.6%) were working in the municipal network of another city in Tocantins and 21 (8.8%) were working in a municipal service outside of the state of Tocantins. Furthermore, 88 (36.7%) said that they were working in a state health department, and 174 (72.4%) said that they were working in a private network. For more details, see Table 1.

Considering the specialties, 50 (20.8%) chose family and community medicine, 47 (19.6%) internal medicine, 41 (17.1%) pediatrics, 39 (16.3%) general surgery, 17 (7.1%) gynecology and obstetrics, 14 (5.8%) anesthesiology, three (1.3%) psychiatry, two (0.8%) orthopedics and traumatology, four (1.7%) neonatology, five (2.1%) vascular surgery, one (0.4%) dermatology, one (0.4%) infectious diseases, four (1.7%) angioradiology, three (1.3%) digestive system surgery, three (1.3%) intensive medicine, four (1.7%) rheumatology and two (0.8%) pediatric intensive care medicine.

It is important to note that family and community medicine has increased greatly over the years, considering that in 2013 and 2015 no residents graduated in this field, and in 2014 only one. However, in 2016 the numbers started to increase. The number of residents in general surgery did not change over the years studied, staying between four and six trained physicians, and this was also seen in relation to gynecology and obstetrics (between three and four) and pediatrics (between five and six).

Orthopedics and traumatology, dermatology, vascular surgery, infectiology, pediatric intensive care medicine and neonatology are more recent specialties in the state of Tocantins, and so the number of trained doctors is still low. Regarding the first four of these programs, the first residents only graduated in 2019, while in the penultimate program, this was in 2017 and in the last program, in 2018. Psychiatry varied over these six years, since in 2013, 2015, 2016 and 2018 there were no resident physicians, while in 2014 and 2017 there was one in each year and in 2019 there were two resident psychiatrists. It can be concluded that there was an increase in the number of specialties over these last years.

### Main outcome: staying in the state of Tocantins

**Figure 1:**
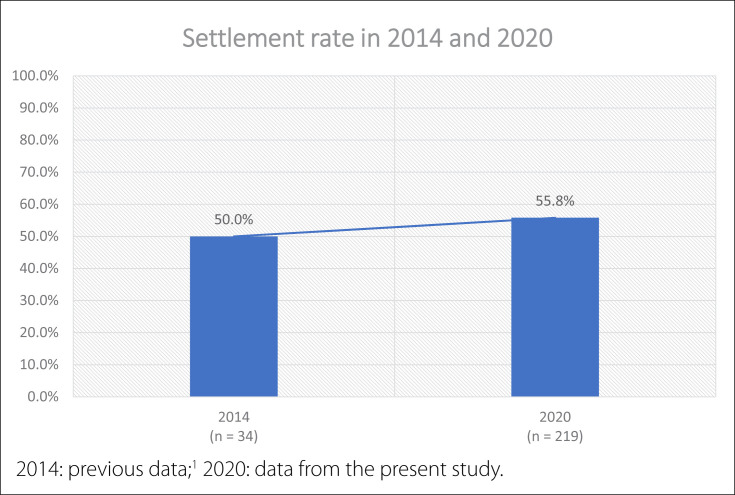
Settlement rates in 2014 and 2020 (years of assessment, i.e. one year after the respective year of occurrence).

Figure 1 presents the percentages of settlement in Tocantins according to the year of conclusion of residency and compares these with previous data (published in 2018, comprising data relating to 2014).^3^ As in the previous study, the figures have been adjusted for those who were not public-sector employees before residency.

In the multivariate logistic regression analysis using the general linear model (GLM) procedure, we used to stay in Tocantins as the dependent variable and other variables as factors. The details are presented in Table 2. Working in the state capital (Palmas) [B = 1.73 ± 0.44; Exp(B) = 5.66 (confidence interval, CI = 2.38-13.50); P = 0.00009] and working in the public network of Palmas [B = 1.73 ± 0.44; Exp(B) = 5.67 (CI = 2.38-13.50); P = 0.00009] were the only significant factors related to staying in the state of Tocantins after finishing medical residency.

**Table 2:** Multivariate analysis by logistic regression using the general linear model procedure

Variable		B (SE)	Exp(B) (CI)	Sig.
**Age up to 30 years**		-0.16 (0.28)	0.85 (0.49-1.49)	0.572
**Age 31-40 years**		-0.17 (0.16)	0.84 (0.62-1.15)	0.275
**Age > 40 years**		-0.81 (0.42)	0.44 (0.19-1.02)	0.056
**Male**		-0.17 (0.22)	0.84 (0.55-1.30)	0.443
**Female**		-0.27 (0.16)	0.76 (0.56-1.05)	0.095
**Concluded in 2013**		-1.01 (0.58)	0.36 (0.12-1.14)	0.083
**Concluded in 2014**		0.49 (0.45)	1.62 (0.67-3.92)	0.280
**Concluded in 2015**		-0.17 (0.41)	0.87 (0.38-1.89)	0.683
**Concluded in 2016**		-0.87 (0.38)	0.42 (0.20-0.87)	0.020
**Concluded in 2017**		-0.10 (0.32)	0.90 (0.49-1.68)	0.752
**Concluded in 2018**		0.04 (0.28)	1.04 (0.60-1.80)	0.889
**Concluded in 2019**		-0.33 (0.27)	0.72 (0.42-1.23)	0.227
**Universidade Federal do Tocantins**		-0.16 (0.15)	0.85 (0.64-1.13)	0.276
**State Health Department of Tocantins**		-0.61 (0.51)	0.54 (0.20-1.47)	0.232
**Municipal Health Department of Palmas**		-0.48 (0.35)	0.62 (0.31-1.24)	0.174
**Single**		0.10 (0.23)	1.11 (0.71-1.74)	0.647
**Married**		0.31 (0.17)	1.36 (0.99-1.89)	0.061
**Divorced**		-0.15 (0.56)	0.86 (0.29-2.55)	0.782
**Widower**		22.57 (0.71)	–	–
**Worked in public network before residency**				
	No	0.06 (0.24)	1.06 (0.67-1.68)	0.814
	Yes	0.31 (0.16)	1.37 (1.00-1.85)	0.046
**Worked in private network before residency**				
	No	0.23 (014)	1.26 (0.95-1.67)	0.113
	Yes	0.25 (0.29)	1.29 (0.73-2.27)	0.388
**If settled down in Tocantins, why?**				
	Family matters	22.57 (0.11)	–	–
	Opportunities in jobs	22.57 (0.19)	–	–
	Demand for specialty	22.57 (0.41)	–	–
	Local identification/ quality of life	22.57 (0.28)	–	–
	Another residency in the state	22.57 (0.38)	–	–
	Work in a field other than medicine	22.57 (1.0)	–	–
**If did not settle down in Tocantins, why?**				
	Family matters	-22.56 (0.26)	–	–
	Issues related to jobs	-3.13 (1.02)	–	–
	Another residency	-22.57 (0.17)	–	–
	Other reasons	-22.57 (0.50)	–	–
**Current municipality**				
	Palmas	1.73 (0.44)	5.66 (2.38-13.50)	0.000090
	Other	1.50 (0.78)	4.50 (0.97-20.82)	0.054
	Out of state	-2.25 (0.74)	0.10 (0.02-0.45)	0.002
**Still works in the field of the specialization**				
	No	0	1.00 (0.59-1.69)	1.000
	Yes	0.31 (0.15)	1.36 (1.01-1.82)	0.040
**Hours worked weekly**				
	Up to 60 hours	-0.27 (0.23)	0.76 (0.48-1,21)	0.246
	More than 60 hours	0.47 (0.16)	1.59 (1.17-2.18)	0.003469
**Bigger salary than in residency**				
	No	-0.13 (0.52)	0.87 (0.32-2.41)	0.796
	Yes	0.27 (0.13)	1.31 (1.00-1.70)	0.046
**Works in a municipal network in the current state**				
	No	0.12 (0.15)	1.13 (0.83-1.52)	0.440
	In the capital	1.73 (0.44)	5.67 (2.38-13.50)	0.000090
	Another city	1.50 (0.78)	4.50 (0.97-20.83)	0.054
	Out of state	-2.25 (0.74)	0.10 (0.02-0.45)	0.002
**Currently works in a private network**				
	No	0.43 (0.25)	1.54 (0.94-2.52)	0.087
	Yes	0.16 (0.15)	1.17 (0.87-1.58)	0.289
**Currently works for a state health department**				
	No	0.29 (0.16)	1.34 (0.97-1.85)	0.075
	Yes	0.14 (0.21)	1.15 (0.75-1.74)	0.523
**Specialty**				
	Internal medicine	-0.39 (0.30)	–	–
	Family and community medicine	0.24 (0.29)	–	–
	Orthopedics and traumatology	0 (1.41)	–	–
	Anesthesiology	0 (0.53)	–	–
	General surgery	-0.58 (0.33)	–	–
	Vascular surgery	22.57 (0.44)	–	–
	Dermatology	-22.57 (1.0)	–	–
	Gynecology and obstetrics	1.18 (0.57)	–	–
	Infectiology	22.57 (1.0)	–	–
	Pediatric intensive care medicine	22.57 (1.0)	–	–
	Neonatology	1.01 (1.15)	–	–
	Pediatrics	0.55 (0.32)	–	–
	Psychiatry	22.57 (0.58)	–	–
	Angioradiology	22.57 (0.50)	–	–
	Digestive system surgery	22.57 (0.58)	–	–
	Intensive medicine	0.69 (1.22)	–	–
	Rheumatology	1.10 (1.15)	–	–
**Did residency improve your medical practice?**				
	No	22.56 (1.00)	–	–
	Yes	0.23 (0.13)	–	–

CI = confidence interval; SE = standard error; Sig. = significance.

## DISCUSSION

In the literature, some data have suggested the possibility that doctors might remain in the places where they did their training.^6^ However, these data vary and the values are not high in all locations.^7–10^

In November 2020, Brazil reached a total of 500,000 doctors, a historic milestone. With this, the country now has a ratio of 2.38 doctors per 1,000 inhabitants. In January 2020, out of the total of 478,010 doctors working in the Brazil, 61.3% had one or more specialist titles, while 38.7% had no title in any specialty.^1^ In absolute numbers, Brazil has 293,064 specialist physicians and 184,946 general practitioners, thus resulting in a ratio of 1.58 specialists for each general practitioner. In addition, the offer of residency vacancies, as well as the presence of specialists, is uneven across regions and states, especially in the northern region.^1^

In previously published data from Brazil, out of 107,114 doctors who graduated from residencies in places other than where they were born, 27,106 (25.31%) were living in the city where they graduated, including some major centers of attraction: approximately 60% of those who stayed where they graduated from residency remained in seven state capitals, of which five were in southeastern Brazil.^11^

Recent data showed that some medical practitioners had changed the Brazilian state in which they lived: 28% were currently in a state other than the one in which they started their careers. Moreover, some were traveling between municipalities to work: one third were working in a city other than the one in which they lived.^1^ However, most doctors (72%) did not change the state at any point during their careers, such that always lived and worked in the same state. Among the other 28% who changed state, more than half (54.9%) moved permanently. The others moved temporarily, possibly for personal reasons, work or professional training.^1^

In 2019, overall in Brazil, 64.5% of doctors were working in the same city where they lived; 27.4% worked in the city where they lived, but also traveled to work in another city; and 8.1% only worked in a city other than where they lived.^1^

Our previous survey considering only the years 2013 and 2014 revealed that 50% of graduated residents remained in the state of Tocantins.^3^ Comparing this with the current data, from which we found that 55.8% remained in this state, the increase was only 5.8%. The reasons for staying that were reported included family matters, work opportunities and doing another residency, and these were like the reasons for not staying. Thus, settlement decisions among new specialists are complex and public-sector managers need to be aware not only of the demand for specialties, but also of personal issues that may influence these specialists, other than job opportunities and good salaries after completion of the residency and the offer of continuity in other local residencies. Across the seven-year period covered by our study, the increase in the settlement rate was small.

In another study, reasons why family doctors remained in their positions were evaluated. The results revealed that there was a high turnover of physicians, figured out by professional dissatisfaction, inadequate working conditions and heavy workload. Among the characteristics of the local human resources policy, distortions in relation to payment stood out, along with problems in the job, career and salary plan relating to family doctors, which limited and penalized the professional's advancement. The main reasons identified in that study that favored permanence were identification with the philosophy of the strategy, professional vocation and the possibility of serving the community.^4^

The geographical distribution and career trajectory of medical graduates and the factors associated with their choice of practice location was evaluated in another study in which a total of 563 graduates completed a questionnaire. Among these, 4.3% reported that family medicine was their medical specialty, 19.9% reported other primary care specialties (internal medicine, pediatrics, surgery and obstetrics-gynecology) and the remainder chose subspecialties. Larger cities were more likely to be chosen for practice, particularly by newly graduated doctors. The job invitations received during medical residency training increased the likelihood of choosing highly populated cities. In contrast, job invitations received during medical school increased the likelihood of choosing less populated cities. Among medical practitioners in cities with a lower population density, proximity to family members was an additional influencing factor, while those who chose more densely populated cities did so because of better infrastructure and recreational options.^12^

In another study, the results showed that the employment attribute that most affected the respondents’ choice was the location of the work, followed by working conditions, payment, access to medical residency, type of contract and workload. It was found that respondents who had attended private colleges, those with higher family income and females generally showed greater resistance to moving to unsafe urban regions and to remote areas of the interior. The employment scenarios that proved to be the most plausible in terms of public intervention were those that combined intermediate wages, good working conditions and obtaining an additional 10 to 20 points in medical residency examinations.^13^ Higher income, satisfaction with training decisions and board certification were also variables associated with a higher retention rate.^14^

## CONCLUSIONS

We see that medical residency led to insertion of new specialists in the state of Tocantins. However, the percentage of residents who remained had only increased by 5.8%, even years after implementation of the first programs. Most specialists are still working for the state health department, with a smaller proportion in municipal health departments, and concentration of specialists in the state capital. We presented reasons for staying in the state that corroborated data in the literature. In the current study, working in the state capital was the only significant factor. Therefore, the reasons that attract specialists to priority areas continue to be diverse and, hence, a variety of measures need to be adopted by public-sector management. The decisions of physicians to stay or leave showed a cost-benefit pattern once their basic needs had been met.^15^
